# What are the neural correlates of meta-cognition and anosognosia in Alzheimer's disease? A systematic review

**DOI:** 10.1016/j.neurobiolaging.2020.06.011

**Published:** 2020-10

**Authors:** Brendan Hallam, Justin Chan, Sergi Gonzalez Costafreda, Rohan Bhome, Jonathan Huntley

**Affiliations:** a Division of Psychiatry, University College London, London, UK; b Camden and Islington NHS Foundation Trust, London, UK

**Keywords:** Alzheimer's disease, Anosognosia, Neural-correlates, Neuroimaging, Systematic review

## Abstract

Awareness of one's own cognitive processes *(metacognition)* or of one's own illness or deficits (*anosognosia*) can be impaired in people with Alzheimer's disease (AD). The neural correlates of anosognosia within AD remain inconclusive. Understanding anosognosia is of importance because of its impact on carer burden and increased institutionalization. A systematic review of structural and functional neuroimaging studies was conducted to identify specific brain regions associated with anosognosia within AD. Thirty-two studies were included in the systematic review. Reduced gray matter density, cerebral blood flow, and hypometabolism in 8 key regions were significantly associated with increased anosognosia scores in people with AD. The most frequently associated regions were the inferior frontal gyrus, anterior cingulate cortex, and medial temporal lobe. Other key regions include the superior frontal gyrus, medial frontal gyrus, orbitofrontal cortex, posterior cingulate cortex, and the insula. Identifying brain regions associated with anosognosia can aid understanding and identification of anosognosia in people with AD and potentially facilitate improvements in care.

## Highlights

• Thirty-two studies included within the systematic review.
• Eight key brain regions were linked with anosognosia within Alzheimer's disease.
• Reduced gray matter density and cerebral blood flow linked with anosognosia.
• More homogenous studies needed to be able to conduct meta-analysis.

## 1 Introduction

### 1.1 Metacognition and anosognosia

Metacognition refers to the “monitoring and control of thought” (Martinez, 2006, p. 696). A related concept is self-awareness, defined as “the realistic perception or appraisal of a given aspect of one's situation, functioning, or performance” (Sunderaraman and Consentino, 2017, pg.2), which has long been studied in the field of cognitive psychology to assess cognition and learning during the developmental stages of healthy young adults. However, in more recent years, there has been an interest in examining metacognition within older adults, especially within clinical populations such as people with Alzheimer's Disease (AD) (Rosen et al., 2014).

In a parallel field of research, clinicians have extensively studied “anosognosia”—a concept first termed by Babinski in 1914 to describe the unawareness of paralysis after a stroke (Babinski, 1914). This term has since gone on to describe unawareness of one's own condition across a range of clinical populations including dementia (Sunderaraman and Consentino, 2017). Although anosognosia is observed in a variety of neurological disorders, it is especially common in AD, where it is often described as lack of insight or awareness of cognitive and functional impairments (Wilson et al., 2016). The frequency of anosognosia is estimated to be between 20% and 80% for people with AD (Castrillo-Sanz et al., 2016; Orfei et al., 2010; Starkstein, 2014). The variability of diagnosing anosognosia within AD is reflected in the large number of anosognosia screening instruments, leading to a lack of specificity in the diagnosis (Mondragón et al., 2019).

### 1.2 Clinical importance of anosognosia

Diagnosing and understanding anosognosia in people with AD is clinically important for several reasons. The presence of anosognosia can worsen medication management (Consentino et al., 2011) and other treatment outcomes (Koltai et al., 2001). Anosognosia is also associated with increased carer burden, social isolation, and stress for both the person with dementia and their caregiver (Seltzer et al., 1997; Turró-Garriga et al., 2013). This in turn increases the likelihood of carer burnout, social services input (Turró-Garriga et al., 2016), and institutionalization for the person with AD (Starkstein et al., 2007). Turró-Garriga et al. (2016) conducted a 24-month longitudinal study on 221 patients evaluating the consequences of anosognosia on total costs of informal care. After adjusting for other factors such as dementia severity and level of support, the presence of anosognosia alone increases the cost of care by over €300/mo on average (Turró-Garriga et al., 2016). Additionally, anosognosia may be a potential independent predictor of conversion from mild cognitive impairment (MCI) to AD (Gerretsen et al., 2017). Therefore, numerous studies have highlighted the importance of improving understanding of anosognosia in people with AD to provide better management and support.

Different methods have been used to investigate lack of insight in AD, but many of the methods can be largely categorized into 3 approaches: (i) clinician rating—where a clinician makes a judgment regarding the patients' levels of insight; (ii) patient-carer discrepancy—where patients and carers independently assess the patients' ability on a function, and the discrepancy scores between patients and carers are used as a measure of the patients' awareness; (iii) self-appraisal performance discrepancy—where patients either prospectively or retrospectively score how well they think they have performed on a measure of cognition, and this is compared with their actual score. The discrepancy between the subjective and actual scores is the measure of anosognosia.

### 1.3 Metacognition

Metacognition has been assessed using a variety of measures, partly depending on the underlying cognitive ability and subtype of metacognition. For example, metamemory is defined as the individual's knowledge of monitoring and control of their own memory processes (Nelson and Narens, 1990). In people with AD, metamemory has typically been assessed using (i) Feeling of Knowing (FoK) judgments, (ii) Judgment of learning (JOLs), and (iii) Remember/Know paradigms (R/K paradigm). These approaches are used in combination with semantic or episodic memory tasks to assess an individual's beliefs about their ability to learn, recall, or recognize information (Consentino and Stern, 2005).

In FoK judgments, individuals are typically asked to learn and recall information and then predict the likelihood that they can subsequently recognize information they had failed to recall earlier (Souchay and Isingrini, 2012). In JOL tasks, the individual is asked to memorize a stimulus (e.g., word pairs) and then asked to predict the likelihood of being able to recall the information correctly. Metrics of metacognitive accuracy can be derived from the discrepancy between the individual's predictions and actual scores. These include the relative accuracy of judgments, reflecting how judgments vary with actual performance (referred to as resolution) or the overall level of overconfidence or underconfidence of the judgments compared with performance (referred to as calibration or metacognitive bias) (Bertrand et al., 2018, Fleming and Lau, 2014). The other common method of assessing metamemory in people with AD is the R/K paradigm. The R/K paradigm differentiates that “remembering” information is a more conscious process of recollection utilizing episodic memory to retrieve key details of the stimuli (Tulving, 1985). On the other hand, “Knowing” involves having a sense of familiarity with the stimuli but less ability to recollect or retrieve the details or context of learning and is typically associated with semantic memory (Tulving, 1985). As stated above, there are distinct differences between definitions of anosognosia and metacognitive (or metamemory), which reflect their respective origins in clinical and healthy populations (Bertrand et al., 2019). While the different methods used to assess anosognosia and metacognition may have inherent biases or reflect different global or specific aspects of metacognition, they all have a common aim to obtain information regarding an individual's knowledge of their own cognitive deficits or illness.

### 1.4 Neural correlates of anosognosia and metacognition in Alzheimer's disease

Many studies investigating the neural correlates associated with anosognosia or loss of insight in AD have produced inconsistent findings because of the use of heterogeneous methodological approaches (Cosentino et al., 2015). The first study that investigated the neural correlates of anosognosia within dementia was carried out by Reed et al. (1993). The study used a simple semistructured interview which classified patients into “full awareness”, “shallow”, and “no awareness”. Using single-photon emission computed tomography (SPECT), Reed et al. (1993) observed that patients with no awareness had significantly lower cerebral blood flow situated in the right dorsolateral frontal lobe compared with patients with full awareness. Since this novel and landmark article, studies have gone on to use a range of both structural and functional imaging techniques to contribute to the understanding of the neural correlates of anosognosia. Structural imaging techniques aim to visualize the anatomical structures of the brain in contrast to functional imaging techniques which use measures of cerebral blood flow or metabolism to assess brain function. For example, 1 technique used in SPECT and PET imaging involves injecting a radioactive tracer to highlight the amount of blood flow to a specific region in the brain within a given time, known as regional cerebral blood flow (rCBF) (Sharma, 2011). rCBF is closely related to another imaging measurement, cerebral metabolic rate of glucose (rCMRglc), which is monitored using fluorodeoxyglucose-positron emission tomography (FDG-PET) scans. rCMRglc directly measures brain function by quantifying regional glucose metabolism (Ma and Eidelberg, 2007). Unlike rCBF and rCMRglc, blood oxygen level–dependent contrast is an indirect noninvasive and radiation-free method used in functional magnetic resonance imaging (fMRI) to measure neural activity and connectivity by proxy (Schultz et al., 2012). Perrotin et al. (2015) investigated if using 2 different neuroimaging modalities produced overlapping neural correlates. Their results demonstrated that the orbitofrontal gyrus and posterior cingulate cortex (PCC) were associated with anosognosia across both fMRI and FDG-PET.

Nevertheless, brain regions identified in previous studies assessing anosognosia in AD remain heterogeneous. Studies have correlated anosognosia scores with regions across the whole brain: frontal (Kashiwa et al., 2005; Vogel et al., 2005), parietal (Tondelli et al., 2018), temporal (Therriault et al., 2018; Tondelli et al., 2018), occipital lobes (Ott et al., 1996; Sultzer et al., 2014), and the cerebellum (Amanzio et al., 2011; Guerrier et al., 2018). There have been previous reviews exploring the overarching topic of neural correlates of anosognosia, but each with distinct criteria and aims. For example, Zamboni and Wilcock (2011) included all types of dementia within their review, whereas Mondragón et al. (2019) focused on functional correlates in MCI and AD. Therefore, there appeared a gap in the literature to provide an up to date systematic review using both structural and functional imaging techniques to outline neural correlates of anosognosia in people with Alzheimer's disease.

### 1.5 Purpose

Aim: To identify and describe the neural correlates of anosognosia within people living with Alzheimer's disease, using both structural and functional imaging techniques.

Objectives: (i) To examine brain regions significantly associated with anosognosia in at least 2 studies using structural imaging techniques, (ii) to examine brain regions significantly associated with anosognosia in at least 2 studies using functional imaging techniques, and (iii) to combine and examine brain regions significantly associated with anosognosia across at least 2 structural studies and 2 functional imaging techniques.

## 2 Methods

### 2.1 Protocol

The authors registered the study protocol with Prospero. Protocol for the systematic review can be found here: https://www.crd.york.ac.uk/prospero/display_record.php?ID=CRD42019137107.

### 2.2 Search strategy

The literature search was conducted using 4 online bibliographic databases (Medline, PsycINFO, EMBASE, Web of Science) on 24th April 2019. Full search terms used for the databases are shown in Supplementary Table 1. Limits for all the bibliographic databases were human studies only, English language only, and original research published in peer-review journals. No time limit was used. Authors were contacted to obtain missing or additional information if necessary. Reference lists of included studies were searched manually to identify potential studies that were not captured in the search as well as key relevant reviews.

### 2.3 Inclusion and exclusion criteria

To be included, studies were required to assess people with AD aged over 65 years. The study must use a neuroimaging method (structural or functional) to assess neural correlates of metacognition/anosognosia. Neuroimaging methods included in the current study are magnetic resonance imaging, diffusion tensor imaging, diffusion-weighted imaging, fMRI, PET, and SPECT scans. Any measurements of anosognosia or tasks involving a metacognitive judgment on awareness, anosognosia, or metacognition were included. Exclusion criteria included people diagnosed with other types of dementia (e.g., frontotemporal dementia) or young onset AD (aged <65). Gray literature, conference posters, reviews, and book extracts were all excluded as these formats would not have been peer-reviewed. Additionally, non-English articles were excluded because of the inability to translate papers in other languages. Animal studies were also excluded because of the focus being on neural correlates of humans with AD.

### 2.4 Data extraction and quality assessment

Studies that did not meet all the eligibility criteria were excluded from the systematic review. Four reviewers were involved in assessing eligibility at the title and abstract stage, with each paper screened by 2 reviewers independently. If either reviewer considered a study potentially relevant, it was retrieved and included into the full text screening. Two reviewers then independently screened eligibility of the full-text studies. Studies that met the inclusion criteria were included in the systematic review. Any discrepancy was resolved using a third independent author.

From the final included studies, data were extracted using a standardized prepiloted data collection form from 2 independent reviewers. Extracted data included author (year), patients, Mini-Mental State Examination, anosognosia measure, stimuli (metacognitive task), type of imaging, threshold of scan, type of analysis, peak coordinates, significant brain regions, Brodmann Area, and correlation direction. Study authors were contacted for any missing data. Once included studies were finalized and data were extracted, a risk of bias assessment was conducted using the Newcastle-Ottawa scale (Anthony and Lin, 2017) to monitor the quality of studies included in the review.

## 3 Results

**Fig. 1:**
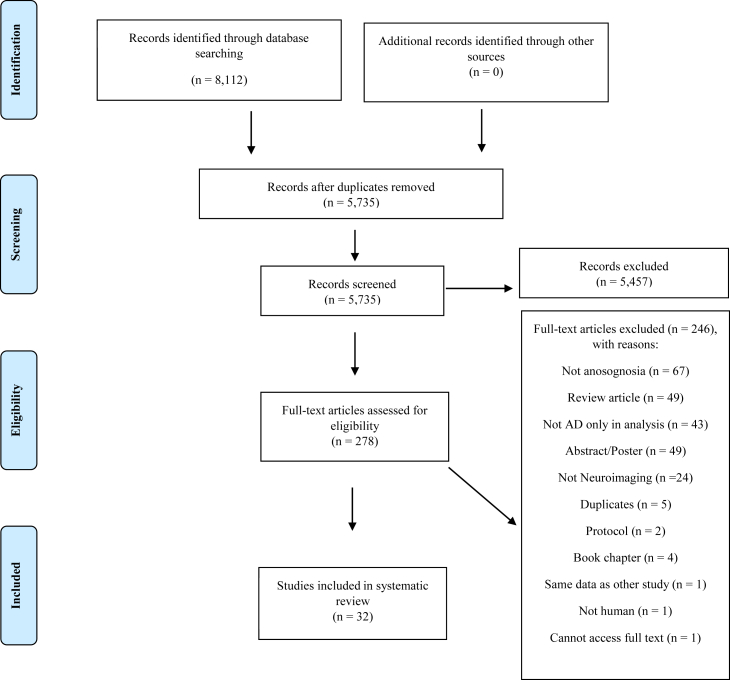
PRISMA flowchart of included studies. Abbreviations: AD, Alzheimer's disease; PRISMA, Preferred Reporting Items for Systematic Reviews and Meta-Analyses.

The search across 4 online bibliographic databases yielded 8112 results. Fig. 1 shows the Preferred Reporting Items for Systematic Reviews and Meta-Analyses flowchart of the selection process for the current review. After duplicate records were removed, a total of 5735 papers were screened by 2 independent reviewers for eligibility based on title and abstract using inclusion and exclusion criteria. Two hundred seventy-eight full-text papers were retrieved to go through the next stage after 5457 papers were excluded for lack of relevance. From the 278 papers full-text screen, 32 articles fit the eligibility criteria and were included in the systematic review. Two hundred forty-six papers were excluded for various reasons, with the most common being no assessment of anosognosia. The inter-rater agreement for full-text screening was *k* = 0.89. The modified Newcastle-Ottawa quality assessment scale for all studies included in the systematic review can be found in Supplementary Table 2.

### 3.1 Structural neuroimaging studies

#### 3.1.1 Study characteristics

Fourteen structural neuroimaging studies form the systematic review (see Table 1). Studies used a range of anosognosia measurements with 3 studies using clinician rating (De Castro et al., 2007; Senturk et al., 2017; Tondelli et al., 2018), 9 using patient-carer discrepancy scores (De Castro et al., 2007; Fujimoto et al., 2017; Guerrier et al., 2018; Hornberger et al., 2014; Perrotin et al., 2015; Philippi et al., 2017; Ruby et al., 2009; Senturk et al., 2017; Tondelli et al., 2018), and zero studies using self-appraisal discrepancy scores without carer verification. For structural studies using metacognitive measurements, 3 used FoK (Bertrand et al., 2018; Consentino et al., 2015; Genon et al., 2016), 3 used Judgment of Learning (Massimo et al., 2013; Perrotin et al., 2015; Tondelli et al., 2018), and 2 used the R/K paradigm (Genon et al., 2014; Phillip et al., 2017). Three studies used more at least 1 anosognosia measurement and 1 metacognitive measurement. See Supplementary Table 3 for specific details of both anosognosia and metacognition measurement used across the structural studies included in the current review. Nine studies provided regions that appear to be significantly associated with anosognosia. Six studies used a whole brain approach, and the other 8 studies used a regions of interest approach, which focuses on specific brain regions considered relevant a priori, with only these regions of interest examined and reported.

**Table 1:** Study characteristics of structural imaging studies included in systematic review

Author (y)	N of patients	MMSE (SD)	Anosognosia measure	Metacognitive stimuli	Type of scan	Threshold	Analysis	Peak coordinate (MNI/T88)			Region [Brodmann area]	Correlation
								x	y	z		
Anosognosia												
De Castro et al. (2007)	21 AD	18.2 (5.0)	Self-Consciousness Questionnaire Denial of illness scale	N/A	CT MRI	ROI	N/A	N/A	N/A	N/A	No significant regions	
Fujimoto et al. (2017)	49 AD	21.6 (2.9)	Anosognosia score	N/A	MRI	Whole brain	Gray matter density	15	18	56	L superior frontal gyrus [6, 8]	↓
Guerrier et al. (2018)	30 AD	24.0 (3.8)	Cognitive Difficulties Scale	N/A	MRI	Whole brain	Gray matter density	−11 0 −53 33	−2 −68 −2 0	42 −27 39 −36	L dorsal anterior cingulate [24] Posterior vermis L postcentral gyrus [6] R fusiform gyrus [20]	↓ ↓ ↓ ↓
Hornberger et al. (2014)	15 AD 24 bvFTD 18 semantic dementia 13 PNFA 11 LPA	22.1 (7.6)	Insight questionnaire	N/A	MRI	ROI	Gray matter density	N/A^✝^	N/A^✝^	N/A^✝^	No significant regions for AD only analysis	
Ruby et al. (2009)	14 AD 17 HC 17 YC	N/A	Judgment of personality	N/A	MRI	Whole brain	N/A	N/A	N/A	N/A	No MRI results reported	
Senturk et al. (2017)	21 AD 26 aMCI	Un-aware 24.1 (2.6) Aware 25.3 (3.1)	Anosognosia Questionnaire Dementia Clinical Insight Rating Scale	N/A	MRI	ROI	N/A	N/A	N/A	N/A	No significant regions found	
Metacognition												
Bertrand et al. (2018)	14 AD 20 HC	23.3 (2.3) 29.3 (1.5)	N/A	Feeling of Knowing (FoK): Episodic retrieval task	MRI	ROI	Cortical thickness	N/A	N/A	N/A	R posterior cingulate cortex R medial prefrontal cortex	↓ ↓
Cosentino et al. (2015)	14 AD 20 HC	23.3 (2.3) 29.3 (1.5)	N/A	Feeling of knowing (FoK): Episodic retrieval task	MRI	ROI	Volumetric ROI	N/A	N/A	N/A	R insula	↓
Genon et al. (2014)	21 AD	23.6 (2.0)	N/A	Adapted Remember/Know paradigm: Word recall Self-recognition task Self-recollection task	MRI	Whole brain	Gray matter density	30 N/A	45 N/A	6 N/A	Lateral prefrontal cortex R/M superior frontal gyrus	↓ ↓
Genon et al. (2016)	23 AD	N/A	N/A	Feeling of Knowing (FoK): Face-name association Episodic and Semantic memory	MRI	Whole brain	Gray matter volume	45 −27 27 −41 N/A 52 31 −33 34 24 46 46 46	27 45 −9 2 N/A −54 −20 −61 −75 −50 28 37 24	13 11 55 39 N/A −14 −22 −44 −44 −49 14 16 31	R inferior/middle frontal L middle orbital gyrus R posterior frontal L precentral gyrus M posterior cingulate cortex R inferior temporal gyrus R medial temporal lobe L cerebellum R cerebellum R cerebellum R inferior frontal gyrus M frontal gyrus M frontal gyrus	↓ ↓ ↓ ↓ ↓ ↓ ↓ ↓ ↓ ↓ ↓ ↓ ↓ ↓
Massimo et al. (2013)	17 AD	20.6 (0.7)	N/A	Judgment of Learning (JOL): Rey Complex Figure Immediate Free Recall	MRI	Whole brain	Gray matter density	34 13 23 −15	49 47 22 37	10 7 40 −30	R frontal pole [10] R Anterior cingulate cortex [32] R Anterior cingulate cortex [32] R Orbitofrontal cortex [11]	↓ ↓ ↓ ↓
Both Anosognosia and Metacognition measures												
Perrotin et al. (2015)	23 AD	21.5 (4.6)	Cognitive Difficulties Scale	Judgment of Learning (JOL): RL/RI-16	MRI	ROI	N/A	N/A	N/A	N/A	No MRI results reported	
Philippi et al. (2017)	1 AD 7 AD 12 HC	25 24.6 (1.0) 28.8 (1.0)	Study specific anosognosia questionnaire	Remember/Know paradigm: Word recognition memory test	MRI	ROI	Gray matter volume	N/A 5^✝^ −5^✝^ 22^✝^ −39^✝^ 40^✝^ −13^✝^ −9^✝^	N/A 59^✝^ 51^✝^ 51^✝^ −12^✝^ −7^✝^ 4^✝^ −101^✝^	N/A −3^✝^ −24^✝^ 12^✝^ −3^✝^ −4^✝^ 19^✝^ 1^✝^	Medial prefrontal cortex R medial frontal gyrus [10] L inferior frontal gyrus (rectus)/orbital [11] R superior frontal gyrus L insula [13] R insula [13] L Caudate L Cuneus [18]	↓ ↓ ↓ ↓ ↓ ↓ ↓ ↓
Tondelli et al. (2018)	12 AD 15 aMCI	23.4 (1.0) 27.3 (0.4)	Anosognosia Questionnaire Dementia Clinical Insight Rating Scale	Judgment of Learning (JOL): Functional Assessment Battery Rey Auditory Verbal Learning Babcock Story Recall Test Rey Figure Recall Stroop	MRI	ROI	Gray matter volume	30^✝^ 26^✝^ −24^✝^ 30^✝^ −34^✝^ N/A N/A	−20^✝^ 62^✝^ −8^✝^ −14^✝^ 20^✝^ N/A N/A	−18^✝^ 20^✝^ −14^✝^ −16^✝^ −26^✝^ N/A N/A	Hippocampus (MTL) Superior frontal gyrus Amygdala (MTL) Hippocampus (MTL) Frontal orbital cortex Anterior cingulate cortex Middle cingulate cortex	↓ ↓ ↓ ↓ ↓ ↓ ↓

All significant regions in studies reached threshold of FWE 0.05.

Key: AD, Alzheimer’s disease; aMCI, amnesic mild cognitive impairment; bvFTD, behavioral frontotemporal dementia; CT, computed tomography; HC, healthy older controls; YC, healthy young controls; L, left hemisphere; LPA, progressive aphasia; M, middle; MMSE = Mini-Mental State Examination; MNI, Montreal Neurological Institute; MRI, magnetic resonance imaging; MTL, medial temporal lobe; PNFA, primary nonfluent aphasia; R, right hemisphere; ROI, region of interest; T88, Talairach; SD = standard deviation.

^✝^ = coordinates published but not AD only.

↓ = negative correlation—as anosognosia score increases, gray matter volume/density decreases.

#### 3.1.2 Structural neural correlates

##### 3.1.2.1 Domains

Similar brain regions were identified across the 3 metacognitive domains: FoK, JOL, and R/K paradigm within structural studies. The frontal gyrus, particularly the superior and inferior frontal gyrus, were identified in FoK (Genon et al., 2016), JOL (Tondelli et al., 2018), and R/K (Genon et al., 2014; Philippi et al., 2017) tasks assessing episodic memory. However, there were some differences such as lower gray matter volume in the anterior cingulate cortex (ACC) being associated with JOL metacognitive measurements (Massimo et al., 2013; Tondelli et al., 2018), but not with FoK or R/K. In contrast, lower gray matter volume in the PCC was associated with FoK metacognitive measurements (Bertrand et al., 2018; Genon et al., 2016), but not with JOL or R/K. Across 9 studies using anosognosia measures, 5 studies reported nonsignificant findings. Of these 5 studies, each study used a patient-carer discrepancy measurement for anosognosia (De Castro et al., 2007; Hornberger et al., 2014; Perrotin et al., 2015; Ruby et al., 2009; Senturk et al., 2017), and 3 used a clinician rated measurement (De Castro et al., 2007; Perrotin et al., 2015; Senturk et al., 2017).

##### 3.1.2.2 Overall

**Fig. 2:**
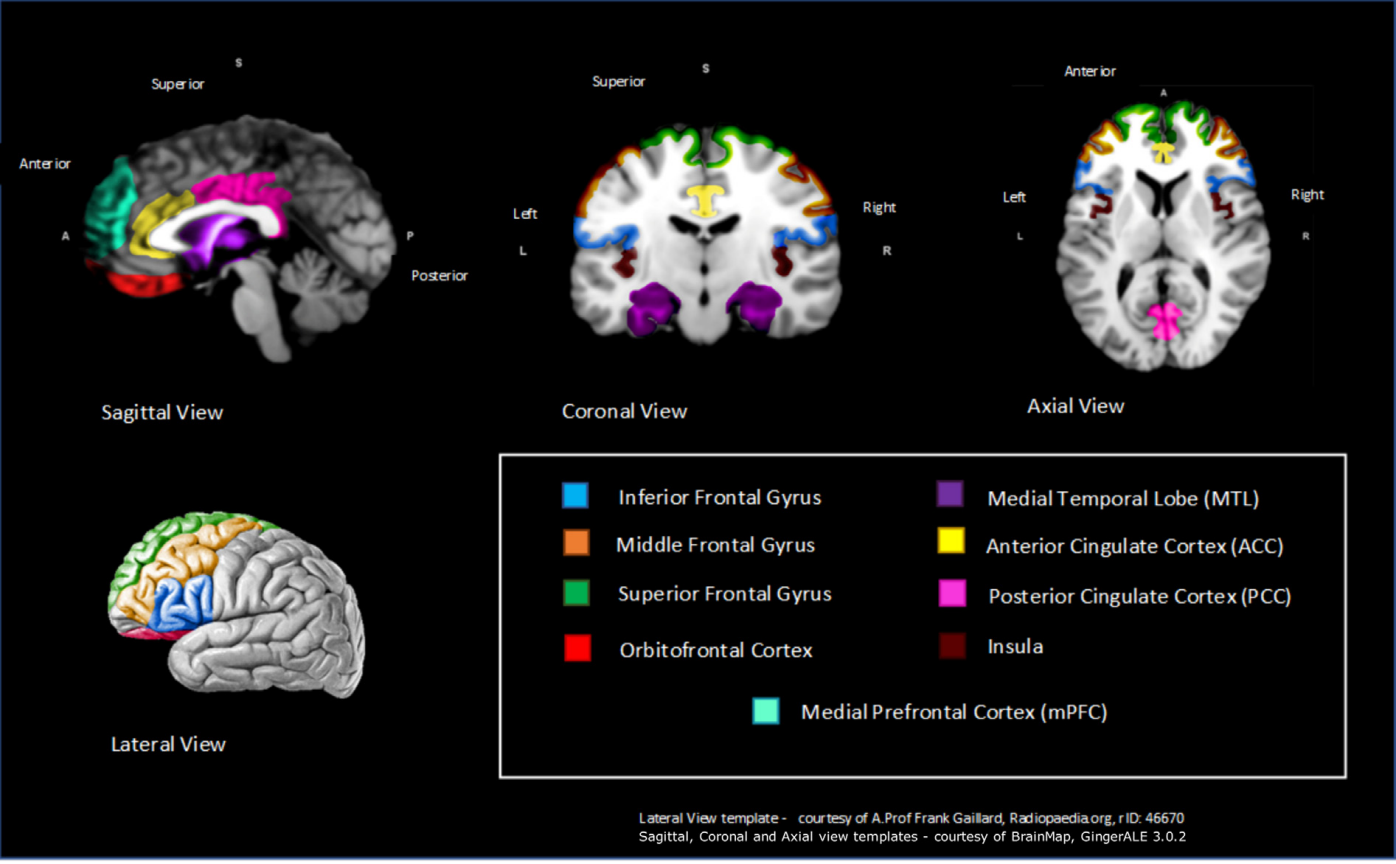
Schematic representation of key brain regions significantly associated with anosognosia or metacognition from at least 2 structural studies.

Higher severity of anosognosia scores were significantly associated with reduced gray matter density/volume in the superior frontal gyrus (Fujimoto et al., 2017; Genon et al., 2014; Philippi et al., 2017; Tondelli et al., 2018), medial temporal lobe (MTL) (Genon et al., 2016; Tondelli et al., 2018), orbitofrontal (Genon et al., 2016; Massimo et al., 2013; Tondelli et al., 2018) medial prefrontal cortex (Bertrand et al., 2018; Philippi et al., 2017), PCC (Bertrand et al., 2018; Genon et al., 2016), ACC (Guerrier et al., 2018; Massimo et al., 2013; Tondelli et al., 2018), and insula (Cosentino et al., 2015; Philippi et al., 2017) (see Table 2). Fig. 2 provides a schematic visual representation of the location of the key brain regions from Table 2.

**Table 2:** Key brain regions with at least 2 structural studies associated with anosognosia or metacognition

Region	N of MRI studies			
Frontal	Anosognosia	Metacognition	Both	Total studies
Inferior frontal Gyrus		1↓	1↓	2↓
Superior frontal Gyrus	1↓	1↓	2↓	4↓
Middle/medial frontal Gyrus		1↓	1↓	2↓
Prefrontal cortex (PFC)				
Orbitofrontal		1↓	2↓	3↓
Medial PFC (mPFC)		1↓	1↓	2↓
Temporal				
Medial temporal lobe		1↓	1↓	2↓
Cingulate cortex				
Anterior cingulate cortex	1↓		2↓	3↓
Posterior cingulate cortex		2↓		2↓
Insular				
Insula		1↓	1↓	2↓

Key: MRI, magnetic resonance imaging.

↓ = negative correlation—as anosognosia/metacognition scores worsen, gray matter volume/density decreases.

### 3.2 Systematic review of functional neuroimaging studies

#### 3.2.1 Study characteristics

Twenty-four functional neuroimaging studies form the systematic review (see Table 3). Twenty-one studies reported regions that are significantly associated with anosognosia. Seven studies used fMRI, 11 used SPECT, and 7 studies used FDG-PET. There were 25 functional analyses as Perrotin et al. (2017) used 2 modalities within 1 study. Twenty studies used a resting-state approach which evaluates function when no explicit task is performed, and 4 studies used an activation approach which evaluates function when an explicit task is performed. Studies used a range of anosognosia measurements with 9 studies using clinician rating (Amanzio et al., 2011; De Castro et al., 2007; Derouesne et al., 1999; Harwood et al., 2005; Ott1996; Reed1993; Sedaghat et al., 2010; Sultzer et al., 2014; Vogel et al., 2005), 15 using patient-carer discrepancy scores (Amanzio et al., 2011; Berlingeri et al., 2015; De Castro et al., 2007; Derouesne et al., 1999; Guerrier et al., 2018; Hanyu et al., 2008; Jedidi et al., 2014; Perrotin et al., 2015; Philippi et al., 2017; Ruby et al., 2009; Salmon et al., 2007; Shibata et al., 2008; Starkstein et al., 1995; Tagai et al., 2018; Zamboni et al., 2013), and zero studies using self-appraisal discrepancy scores without carer verification. For functional studies using metacognitive measurements, zero used FoK, 3 used Judgment of Learning (Berlingeri et al., 2015; Mimura and Yano, 2006; Perrotin et al., 2015), and 3 used the Remember/Know paradigm (Genon et al., 2014; Phillip et al., 2017; Rauchs et al., 2007). Three studies included at least 1 anosognosia and 1 metacognitive measurement. See Supplementary Table 4 for specific details of measurements used across the included functional studies. Twelve studies published coordinates of areas meeting statistical significance.

**Table 3:** Study characteristics of functional imaging studies included in systematic review

Author (year)	N of patients	MMSE (SD)	Anosognosia measure	Stimuli (meta-cognitive)	Type of scan	Threshold	Analysis	Peak coordinate (MNI/T88)			Region [Brodmann area]	Correlation
								x	y	z		
Anosognosia												
Amanzio et al. (2011)	28 AD	22.4 (2.1)	Anosognosia Questionnaire Dementia Clinical Insight Rating Scale	N/A	fMRI (activation)	Whole Brain	BOLD	−7 47 41 10 8 −42 −54 −57 22 −11 −3 26	54 −23 −71 36 23 10 3 13 4 2 −46 −53	5 49 25 3 26 −34 −31 −16 3 1 −36 29	Bilateral rostral prefrontal cortex [10] R postcentral gyrus [2] R middle temporal gyrus [39] R anterior cingulate [24] R anterior cingulate [24] L inferior temporal gyrus [21] L medial temporal gyrus [21] L superior temporal gyrus [38] R putamen L medial globus pallidus Bilateral cerebellum, posterior lobe R cerebellum, anterior lobe	↓ ↓ ↓ ↓ ↓ ↓ ↓ ↓ ↓ ↓ ↓ ↓ ↓
De Castro et al. (2007)	21 AD	18.2 (5.0)	Self-Consciousness Questionnaire Denial of Illness Scale	N/A	HMPAO–SPECT (resting)	ROI	Cerebral perfusion	N/A	N/A	N/A	No significant regions	
Derouesne et al. (1999)	88 AD	22.5 (3.2)	Cognitive Difficulties Scale Clinical interview Psycho- behavioral questionnaire	N/A	123I-IMP SPECT (resting)	ROI	Cerebral perfusion	N/A	N/A	N/A	R frontal	↓
Guerrier et al. (2018)	28 AD	24 (3.8)	Cognitive Difficulties Scale	N/A	FGD-PET (resting)	Whole brain	18F-FDG metabolism	−8	2	38	L dorsal anterior cingulate [24]	↓
Hanyu et al. (2008)	38 AD	Un-aware 25.8 (1.3) Aware 25.5 (1.1)	Everyday Memory Checklist	N/A	123I-IMP SPECT (resting)	ROI	Cerebral perfusion	N/A	N/A	N/A	R/L subcallosal R/L anterior cingulate R cingulate gyrus L orbital gyrus	↓ ↓ ↓ ↓
Harwood et al. (2005)	41 AD	19.3 (6.7)	Neuro-behavioral rating scale (item score)	N/A	FDG-PET (resting)	ROI	18F-FDG metabolism	N/A	N/A	N/A	R superior dorsolateral prefrontal cortex [6] R lateral frontal [8] R superior frontal pole [9] R caudal anterior cingulate [24] R lateral frontal [45]	↓ ↓ ↓ ↓ ↓
Jedidi et al. (2014)	37 AD 25 HC	N/A	Judgment of personality	N/A	FDG-PET (resting)	ROI	18F-FDG metabolism	12 −6 −8 −5^✝^ −5^✝^ −24^✝^	56 54 60 40^✝^ 54^✝^ 44^✝^	20 16 8 28^✝^ 14^✝^ 26^✝^	Dorsomedial PFC Dorsomedial PFC Ventromedial PFC Dorsomedial PFC Dorsomedial PFC Superior frontal sulcus	↓ ↓ ↓ ↓ ↓ ↓
Ott et al. (1996)	40 AD	18.5 (5.5)	Clinical Insight Rating Scale Instrumental Activities of Daily Living	N/A	HMPAO-SPECT (resting)	ROI	Cerebral perfusion	N/A	N/A	N/A	R posterior temporal-occipital cortex L inferior frontal lobe	↓ ↓
Reed et al. (1993)	57 AD	N/A	Anosognosia score	N/A	123I-SPECT (resting)	ROI	Cerebral perfusion	N/A	N/A	N/A	R dorsolateral frontal cortex	↓
Ruby et al. (2009)	14 AD 17 HC 17 YC	N/A	Judgment of personality	N/A	fMRI (activation)	Whole brain	BOLD	−22 −26 40	−66 −50 −48	40 42 38	Intraparietal sulcus Intraparietal sulcus Intraparietal sulcus	↓ ↓ ↓
Salmon et al. (2006)	206 AD	21.0 (4.5)	Self-made anosognosia questionnaire	N/A	FDG-PET (Resting)	ROI	18F-FDG metabolism	22 −18 12 42 −18 40	−12 36 32 6 38 4	−30 −16 −16 0 44 20	R parahippocampal cortex L orbitofrontal cortex R gyrus rectus (inferior frontal gyrus) R insula L superior frontal sulcus R medial temporal lobe/cortex	↓ ↓ ↓ ↓ ↓ ↓
Sedaghat et al. (2010)	42 AD	Un-aware 18.0 (4.0) Aware 21.0 (4)	Clinical interview	N/A	HMPAO-SPECT (resting)	ROI	Cerebral perfusion	N/A	N/A	N/A	R prefrontal lobe R inferior parietal R/L medial temporal lobe/cortex	↓ ↓ ↓
Shibata et al. (2008)	29 AD	21.2 (2.9)	Anosognosia score	N/A	123I-SPECT (resting)	ROI	Cerebral perfusion	−8	38	0	L orbitofrontal cortex	↓
Starkstein et al. (1995)	46 AD	Un-aware 19.1 (5.6) Aware 18.5 (4.7)	Anosognosia Questionnaire Dementia	N/A	HMPAO-SPECT (resting)	ROI	Cerebral perfusion	N/A	N/A	N/A	R frontal inferior gyrus R frontal superior gyrus	↓ ↓
Sultzer et al. (2014)	88 AD	N/A	Neuro-behavioral rating scale (insight item score)	N/A	FDG-PET (resting)	ROI	18F-FDG metabolism	4	54	6	R frontal cortex [8, 9, 10, 24, 32]	↓
Tagai et al. (2018)	37 AD 12 HC	20.4 (4.6)	Anosognosia questionnaire dementia	N/A	123I-SPECT (resting)	ROI	Cerebral perfusion	56 −22 −18 −22 −38 20 46 22 16	36 −46 −50 −50 −22 −36 −82 −16 −98	−6 36 20 20 −22 46 30 50 22	R triangular inferior frontal gyrus L superior parietal lobe L precuneus L posterior cingulate cortex/gyrus L fusiform gyrus R precuneus R middle occipital gyrus R precentral gyrus R occipital lobe	↓ ↑ ↑ ↑ ↑ ↑ ↑ ↑ ↑ ↑
Vogel et al. (2005)	36 AD 30 aMCI 33 HC	24.0 (2.5) 26.1 (2.1) 29.3 (0.9)	Anosognosia rating scale Memory questionnaire	N/A	HMPAO-SPECT (resting)	ROI	Cerebral perfusion	N/A	N/A	N/A	R inferior frontal cortex	↓
Zamboni et al. (2013)	17 AD 17 aMCI 17 HC	22.2 (3.0) 26.8 (1.4) 29.9 (0.7)	Anosognosia questionnaire dementia Patient/carer discrepancy score: Anderson trait list	N/A	fMRI (activation)	Whole brain	BOLD	−4^✝^ −4^✝^ −19^✝^ −6^✝^ −10^✝^ −4^✝^ −42^✝^ −42^✝^ −62^✝^ −62^✝^ −62^✝^ −58^✝^	24^✝^ 8^✝^ 14^✝^ 38^✝^ −2^✝^ 24^✝^ −12^✝^ −16^✝^ −14^✝^ −14^✝^ −12^✝^ −4^✝^	36^✝^ 48^✝^ 48^✝^ 26^✝^ 48^✝^ 48^✝^ −14^✝^ −4^✝^ −20^✝^ −26^✝^ −12^✝^ −14^✝^	L paracingulate gyrus L paracingulate gyrus L paracingulate gyrus L anterior cingulate cortex/gyrus L anterior cingulate cortex/gyrus L superior frontal gyrus L superior temporal gyrus L superior temporal gyrus L middle temporal gyrus L middle temporal gyrus L superior temporal sulcus L superior temporal sulcus	↓ ↓ ↓ ↓ ↓ ↓ ↓ ↓ ↓ ↓ ↓ ↓ ↓
Metacognition												
Genon et al. (2014)	21 AD 21 HC	23.6 (2.0)	N/A	Adapted Remember/Know paradigm: Word recall Self-recognition task Self-recollection task	fMRI (activation)	Whole brain	BOLD	N/A	N/A	N/A	No significant regions identified	
Mimura and Yano (2006)	24 AD	22.3 (3.8)	N/A	Judgment of Learning (JOLs): Auditory verbal learning test	SPECT (resting)	ROI	Cerebral perfusion	0 8 38	57 −48 25	−16 48 1	Medial frontal gyrus/lobe R precuneus Inferior frontal gyrus	↓ ↓ ↓
Rauchs et al. (2007)	13 AD 10 HC	24.8 (2.4) 29.4 (1)	N/A	Remember/Know paradigm: Word recall	FDG-PET (resting)	Whole brain	18F-FDG metabolism	−32^✝^ 30^✝^ 14^✝^ −48^✝^	54^✝^ 54^✝^ 50^✝^ 46^✝^	2^✝^ 10^✝^ 44^✝^ −14^✝^	L middle frontal gyrus [10] R middle frontal gyrus [10] R median superior frontal gyrus [8] L frontal inferior orbital gyrus [47]	↓ ↓ ↓ ↓
Both Anosognosia and Metacognition												
Berlingeri et al. (2015)	18 AD 15 HC	Un-aware 24.1 (2.8) Aware 25.5 (3.4) 28.8 (1.2)	Anosognosia Questionnaire Dementia	Judgment of Learning (JOLs): Episodic word recognition task Semantic Verbal Fluency Test	fMRI (resting)	ROI	BOLD	−39^✝^ −39^✝^	−15^✝^ 12^✝^	−15^✝^ 3^✝^	L insular cortex L middle hippocampus (MTL)	↓ ↓
Perrotin et al. (2015)	23 AD	21.5 (4.6)	Cognitive Difficulties Scale Self-appraisal discrepancies	Judgment of Learning (JOL): RL/RI-16	fMRI (Resting) FDG-PET	ROI	BOLD 18F-FDG metabolism	N/A	N/A	N/A	Medial temporal lobe Orbitofrontal Posterior cingulate cortex Orbitofrontal Posterior cingulate cortex	↓ ↓ ↓ ↓ ↓
Philippi et al. (2017)	1 AD 7 AD 12 HC	25 24.6 (1.0) 28.8 (1.0)	Study specific anosognosia questionnaire	Remember/Know paradigm: Recognition memory test	fMRI (resting)	ROI	BOLD	N/A	N/A	N/A	No fMRI results reported	

All significant regions in studies reached threshold of FWE 0.05.

Key: AD, Alzheimer’s disease; aMCI, amnesic mild cognitive impairment; BOLD, Blood oxygen level–dependent imaging; FDG-PET, fluorodeoxyglucose-positron emission tomography; fMRI, functional magnetic resonance imaging; HMPAO, technetium-99m HMPAO isotope; HC, healthy older controls; L, left hemisphere; LPA, progressive aphasia; M, middle; MMSE, Mini-Mental State Examination; MNI, Montreal Neurological Institute; MRI, magnetic resonance imaging; MTL, medial temporal lobe; PNFA, primary nonfluent aphasia; R, right hemisphere; ROI, region of interest; T88, Talairach; SD, standard deviation; SPECT, single-photon emission computed tomography; YC, healthy young controls;123I, Iodine-123 isotope; 18F-FDG metabolism, Fludeoxyglucose metabolism.

Cerebral perfusion = regional cerebral function.

Resting state = evaluate function when no explicit task is performed.

Activation = evaluate function when explicit task is performed.

Bilateral = Both hemispheres.

↓ = negative correlation—decreased blood flow/glucose.

Metabolism associated with higher anosognosia score.

↑ = positive correlation—increased blood flow/glucose.

Metabolism associated with higher anosognosia score.

✝ = coordinates published but not AD only.

#### 3.2.2 Functional neural correlates

##### 3.2.2.1 Domains

Across functional studies, different brain regions were identified across 2 metacognitive domains, judgment of learning, and remember/know paradigm. Two of the 3 studies using the R/K paradigm did not find any significant brain regions associated with metacognitive impairment, within an AD population. However, within studies using judgment of learning measurements, there was some consistency in identifying the medial temporal lobe (Berlingeri et al., 2015; Perrotin et al., 2015) and frontal gyrus, specifically around the inferior frontal and orbitofrontal gyrus. Across anosognosia measurements, the brain regions identified were similar. Reduced cerebral blood flow and hypometabolism in the inferior frontal gyrus were associated in clinician rated (Ott1996; Vogel et al., 2005) and patient-carer rated measurements (Salmon et al., 2007; Starkstein et al., 1995; Tagai et al., 2018). Similarly, the ACC was associated with both clinician-rated (Amanzio et al., 2011; Harwood et al., 2005) and patient-carer measurement of anosognosia (Amanzio et al., 2011; Guerrier et al., 2018; Hanyu et al., 2008; Zamboni et al., 2013).

##### 3.2.2.2 Overall

**Fig. 3:**
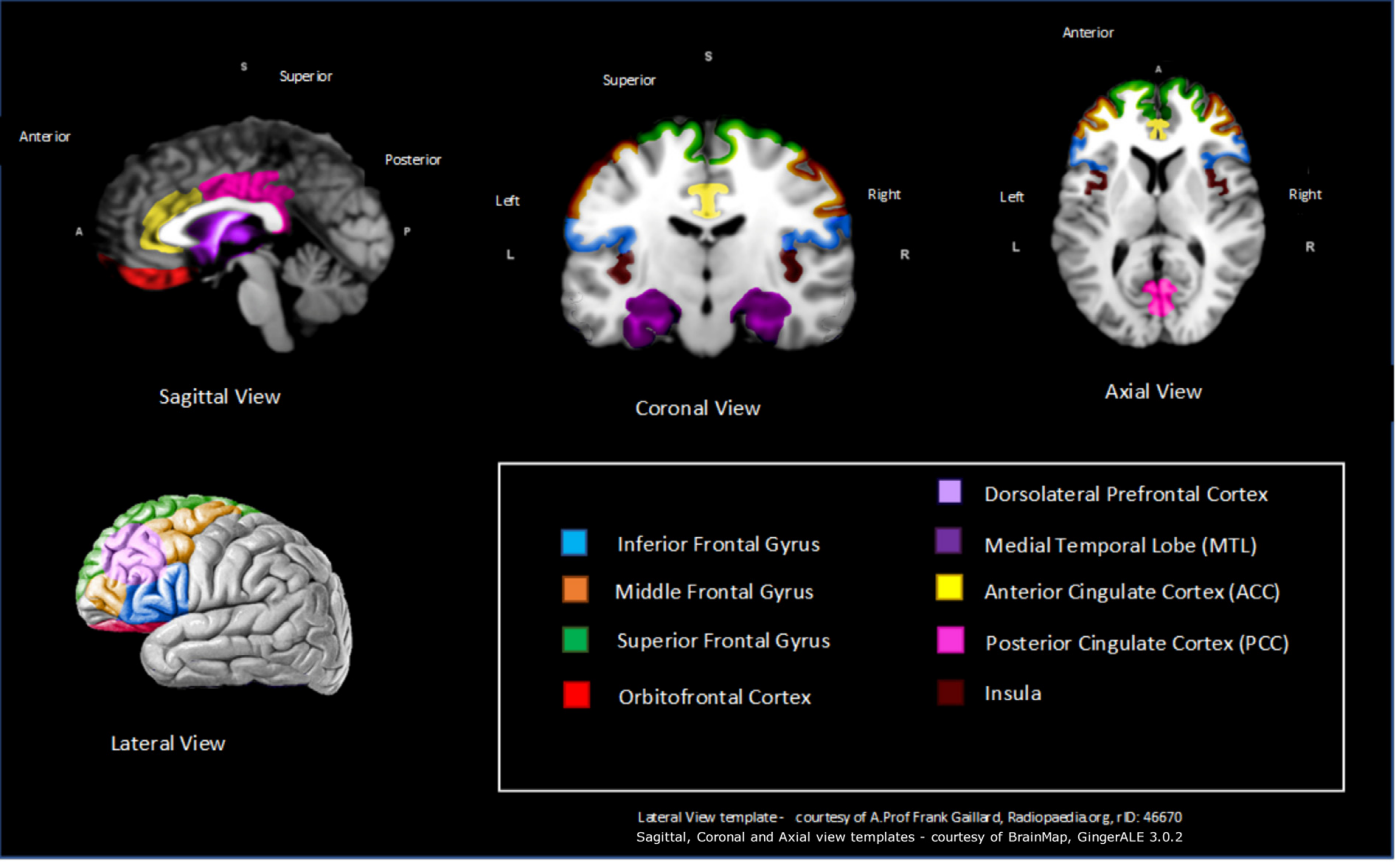
Schematic representation of key brain regions significantly associated with anosognosia from at least 2 functional modalities.

Regions of the brain associated with anosognosia and metacognition are summarized in Table 4. SPECT and FDG-PET scans demonstrated rCBF and hypometabolism in the inferior frontal gyrus (Mimura and Yano., 2006; Ott et al., 1996; Rauchs et al., 2007; Salmon et al., 2007; Starkstein et al., 1995; Tagai et al., 2018; Vogel et al., 2005), superior frontal gyrus (Rauchs et al., 2007; Starkstein et al., 1995; Zamboni et al., 2013), orbitofrontal cortex (Perrotin et al., 2015; Salmon et al., 2006; Shibata et al., 2008), and dorsolateral prefrontal cortex (Harwood et al., 2005; Reed1993) primarily in resting-state acquisitions. Increased anosognosia scores were associated with decreased rCBF and hypometabolism in the MTL (Berlingeri et al., 2015; Perrotin et al., 2015; Salmon et al., 2006; Sedaghat et al., 2010) and ACC (Amanzio et al., 2011; Guerrier et al., 2018; Hanyu et al., 2008; Harwood et al., 2005; Zamboni et al., 2013) across all 3 modalities (fMRI, SPECT, and FDG-PET). Hypometabolism was also associated with higher anosognosia scores within the PCC using FDG-PET (Perrotin et al., 2015). Interestingly however, Tagai et al. (2018) is the only study within the systematic review to suggest that hyperperfusion (higher rates of CBF) is linked with higher rates of anosognosia within the PCC. Tagai et al. (2018) also suggest hyperperfusion happens over 6 regions of the brain, although no other study within the review validates this proposal. The final region where higher anosognosia scores were associated with lower CBF, and hypometabolism was the insula (see Fig. 3).

**Table 4:** Brain regions significantly associated with anosognosia across 2 or more functional modalities

Region	N of fMRI studies				N of SPECT studies				N of FDG-PET studies			
Frontal	A	M	B	T	A	M	B	T	A	M	B	T
Inferior frontal Gyrus					4		1	5↓	1	1		2↓
Superior frontal Gyrus			1	1↓	1			1↓		1		1↓
Middle/medial frontal Gyrus							1	1↓		1		1↓
Prefrontal cortex (PFC)												
Orbitofrontal			1	1↓✝	1			1↓✝	1		1	2↓✝
Dorsolateral PFC					1			1↓✝	1			1↓
Temporal												
Medial temporal lobe			2	2↓	1			1↓	1			1↓
Cingulate cortex												
Anterior cingulate cortex	1		1	2↓	1			1↓	2			2↓
Posterior cingulate cortex			1✝	1↓✝	1			1↑			1✝	1↓✝
Insular												
Insula			1	1↓					1			1↓
None												
None		1	1	2	1			1				

A = Anosognosia study, M = Metacognition study, B = Study using both anosognosia and metacognition measures, T = Total N of studies.

Key: FDG-PET, fluorodeoxyglucose-positron emission tomography; fMRI = functional magnetic resonance imaging

↓ = negative—decreased blood flow/glucose metabolism associated with worse anosognosia/metacognition scores.

↑ = positive correlation—increased blood flow/glucose metabolism associated with worse anosognosia/metacognition score.

✝ Perrotin et al. (2015) found significance across FDG-PET & fMRI.

### 3.3 Comparison of structural and functional studies

**Fig. 4:**
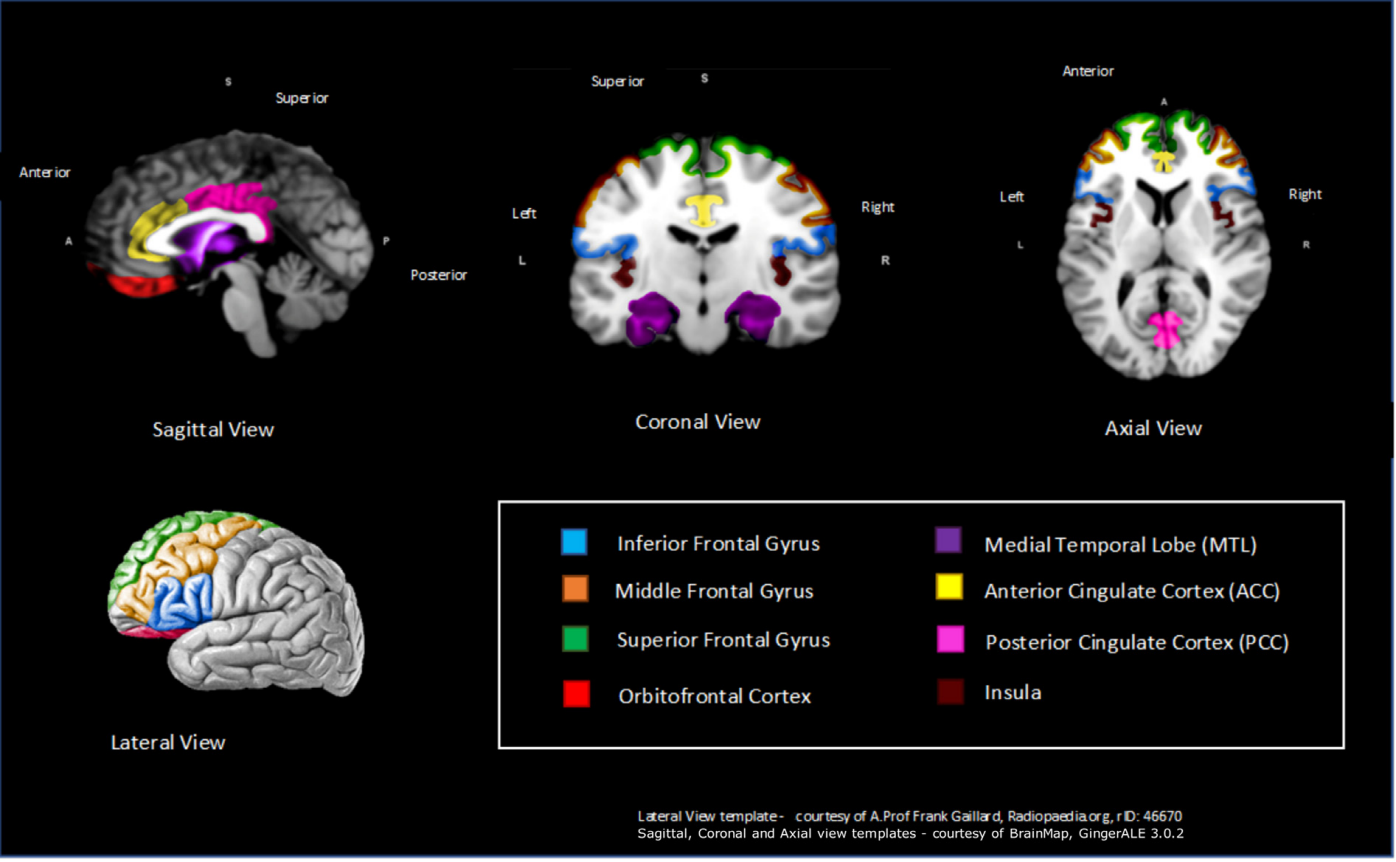
Schematic representation of overall key brain regions significantly associated with anosognosia—across at least 2 structural and 2 functional studies.

Forty-two regions across the brain were significantly associated with anosognosia in at least 1 of the 32 studies, with 8 regions significantly associated with anosognosia in at least 2 structural and 2 functional studies (see Table 5 and Fig. 4). The most frequent brain regions associated with anosognosia across structural and functional imaging are the inferior frontal gyrus, ACC, and MTL. This indicates that higher gray matter atrophy and reduced functional activity within the inferior frontal gyrus, ACC, and MTL is correlated with higher severity of anosognosia. In addition to the inferior frontal gyrus, decreased gray matter density (GMD) and functional activity is associated with other frontal lobe regions including the superior frontal gyrus, middle frontal gyrus, and orbitofrontal cortex. Similarly, reduced functional activity and gray matter of the anterior and PCC are correlated with increased severity of anosognosia. Interestingly however, Tagai et al. (2018) propose that hyperperfusion is linked with higher rates of anosognosia within the PCC. The insula is a small region of the cerebral cortex located within the lateral sulcus and reduced GMD and functional activity in this region is also correlated with increased severity of anosognosia (Berlingeri et al., 2015; Cosentino et al., 2015; Philippi et al., 2017; Salmon et al., 2006).

**Table 5:** Brain regions with at least 2 structural and 2 functional studies correlating with anosognosia

Region	N of structural studies				N of functional studies			
Frontal	A	M	B	T	A	M	B	T
Inferior frontal Gyrus		1	1	2↓	5	1	1	7↓
Superior frontal Gyrus	1	1	2	4↓	2		1	3↓
Middle/medial frontal Gyrus		1	1	2↓		1	1	2↓
Prefrontal cortex (PFC)								
Orbitofrontal		1	2	3↓	2		2	4↓✝
Temporal								
Medial temporal lobe		1	1	2↓	2		2	4↓
Cingulate cortex								
Anterior cingulate cortex	1		2	3↓	3		2	5↓
Posterior cingulate cortex		2		2↓	1		2	3↓/↑✝
Insular								
Insula		1	1	2↓	1		1	2↓

A, anosognosia study, M, Metacognition study, B, Study using both anosognosia and metacognition measures, T, Total N of studies.

↓ = negative—decreased blood flow/glucose metabolism associated with worse anosognosia/metacognition scores.

↑ = positive correlation—increased blood flow/glucose metabolism associated with worse anosognosia/metacognition score.

✝ Perrotin et al. (2015) found significance across FDG-PET & fMRI.

## 4 Discussion

### 4.1 Summary of findings

This comprehensive systematic review of 32 studies has found evidence of the association between certain brain regions and anosognosia. Reduction in gray matter density, cerebral blood flow, or metabolism in 8 key regions of the brain were associated with anosognosia in at least 2 different functional modalities and in at least 2 structural studies. These 8 regions were the inferior frontal gyrus, ACC, MTL, superior frontal gyrus, medial frontal gyrus, orbitofrontal cortex, insula, and the PCC (Fig. 4). Zamboni and Wilcock (2011) have suggested that some of these regions (orbitofrontal cortex, insula, and MTL) are associated with the cognitive processing of self and form part of the default mode network (DMN). The DMN is a large-scale network that comprises of interconnected brain regions that is hypothesized to be associated with functions that have been highlighted as important in self-related cognition, including the ability to imagine future events (Buckner et al., 2008; Weiler et al., 2016). Additional studies support the theory that complex connections within the DMN, such as the PCC and MTL may be key regions of the brain and that if damaged, could increase anosognosia-based symptoms in people with AD (Mevel et al., 2011; Therriault et al., 2018). Antoine et al. (2016) used connectivity analysis of resting fMRI to demonstrate that anosognosia was related to decreased connectivity between the PCC, MTL, and lateral temporal cortex region.

As with any complex phenomena, such as self-awareness, it is not just 1 region that is solely responsible but rather a network of connected regions. Some regions are spatially adjoining such as those in the frontal lobe that appear to be associated with anosognosia, including the inferior frontal gyrus, middle frontal gyrus, and orbitofrontal cortex. Within the cingulate cortex, reduced function in the ACC is the most frequently associated region with anosognosia scores in people with AD across MRI (Guerrier et al., 2018), fMRI (Amanzio et al., 2011; Zamboni et al., 2013), SPECT (Hanyu et al., 2008), and FDG-PET (Guerrier et al., 2018). ACC is located in the middle of brain and has extensive connections between the prefrontal cortex and the rest of the cingulate cortex (Northoff et al., 2006). Across previous studies in healthy controls, the ACC is commonly implicated in self-awareness (Lou et al., 2017). Some studies assert that the PCC regulates balance between internal and external-focused attention, indicating the PCC as an imperative structure in awareness (Leech and Sharp, 2013). It is important to note that the PCC forms a central node of the DMN (Leech and Sharp, 2012). Seven studies highlighted the MTL, as a key region associated with anosognosia scores. This is perhaps unsurprising given the hippocampus is one of the first regions of the brain to atrophy within AD and plays a vital role in learning and memory (Mu and Gage, 2011). Another region that appears to influence self-awareness is the insula, which is highly connected to the amygdala and cingulate cortex with widely different functions including pain perception and processing of social emotions (Nieuwenhuys, 2012). Craig and Craig (2009) have suggested that the insula may be a key region of the brain for awareness, receiving information regarding the location and condition of our bodies, our environment and subjective emotion.

To further understand the mechanisms of self-awareness, Joensson et al. (2015) investigated the potential role of neurotransmitters, specifically dopamine, in the regulation of metacognition. The magnetoencephalography study randomly allocated young healthy age-matched participants into placebo group or dopaminergic stimulation (L-dopa) group. Results suggested that increased (L-dopa) system increased self-awareness and metacognition during tasks. Self-awareness is hypothesized to be regulated by dopamine through the medial prefrontal cortex and ACC via the GABA system (Lou et al., 2011a, Lou et al., 2011b). Dopaminergic innervation in the medial prefrontal cortex, ACC, and right insula (Lou et al., 2017) may therefore be an important mechanism for the strong association between anosognosia, awareness and the insula, ACC, and the prefrontal cortex found in the current review.

The findings identified within the review supports the current theoretical landscape of anosognosia in AD. Lenzoni, Morris and Mograbi (2020) proposed a simplified version of the Cognitive Awareness Model (CAM) in relation to the concept of self. Many key brain regions, such as ACC, frontal lobes, MTL, prefrontal cortex, and insula, are identified as important in both the current review and the CAM. The CAM (Morris and Mograbi, 2013) is designed to outline the process of self-monitoring and self-regulation, which if impaired can lead to a lack of self-awareness or anosognosia. Furthermore, this review is in keeping with other studies that suggest that cortical midlines structures and the DMN could potentially play an important role for processing self-related information (Weiler et al., 2016).

### 4.2 Limitations

One limitation of the literature is the variability of the measurements used to capture anosognosia, which may not be conceptually capturing the same construct of “anosognosia” or metacognition. Within this review alone, there were 17 different measures of anosognosia or metacognition across 32 studies. Therefore, arguably they could be measuring different aspects of anosognosia or metacognition, and thus, it may be difficult to draw clear conclusions about anosognosia in dementia. However, the vast majority of anosognosia measures can be categorized as either patient-carer discrepancy or clinician rating. Additionally, the metacognitive measures can largely be categorized as FoK, JOL, or R/K paradigm. So, while there is large heterogeneity between the study measurements, a strength of this review is identifying areas of the brain that are consistently reported across a range of approaches to provide a detailed overview of brain regions associated with anosognosia and metacognitive impairment in people with AD. Other limitations of the review include only selecting English language studies, excluding gray literature and not having access to certain articles that may be relevant.

### 4.3 Future research

It is of importance in future studies to create a more consistent and objective approach to assessing metacognition and anosognosia. In addition, future research should investigate the neural correlates of anosognosia in AD in relation to progression of disease severity over time. This could be investigated by comparing severity groups in a cross-sectional approach or longitudinally to monitor changes in the brain alongside progression of anosognosia or metacognitive impairments.

### 4.4 Conclusion

Overall, the current review provides an updated synthesis of both structural and functional studies and identifies brain regions associated with anosognosia in AD. Despite the issue of heterogeneity in measuring the complex phenomenon of self-awareness, 8 key regions appear across multiple studies to correlate with anosognosia within the systematic review. Decreased gray matter density, reduced cerebral blood flow, and hypometabolism within the superior frontal gyrus, inferior frontal gyrus, middle frontal gyrus, orbitofrontal cortex, ACC, PCC, MTL, and insula were all identified across both structural and functional studies to correlate with increased severity of anosognosia. Identifying key regions of the brain associated with anosognosia can help to improve our understanding of this important feature of dementia. The use of neuroimaging may aid the assessment of insight and awareness in people with dementia to not only improve our understanding of the effects of the illness but aid assessment and management of anosognosia to reduce the risk of dangerous behaviors, reduce burden, and delay institutionalization.

## Disclosure statement

The authors have no actual or potential conflicts of interest.
