# Supplementary material for: What are the neural correlates of meta-cognition and anosognosia in Alzheimer's disease? A systematic review
Source: Neurobiol Aging. 2020 Oct;94:250–64. doi: 10.1016/j.neurobiolaging.2020.06.011 (PMC7903321; doi:10.1016/j.neurobiolaging.2020.06.011)
Supplement: Supplementary Table 1 [file mmc1.docx]

**Supplementary Materials**

*Supplementary table 1 – Keyword search terms used across bibliographic databases (PsycINFO, EMBASE, Medline, Web of Science)*

| Concept | Search terms |
| --- | --- |
| Concept 1:  Alzheimer’s Disease | *dement* or Alzheimer* or AD or DAT* |
| Concept 2:  Anosognosia/metacognition | *metacognition or metacog* or metamemory or “decision confidence” or “memory confidence” or “feeling of knowing” or “judgement of learning” or conscious* or aware* or insight or anosognosia or “self appraisal” or “self agency” or “self reflect*” or “self referen*”* |
| Concept 3:  Neuroimaging | *neuroimaging or imag* or MRI or “magnetic resonance imaging” or fMRI or PET or “positron emission tomography” or SPECT or “Single-photon emission computed tomography” or CT or “Computed tomography” or “Diffusion tensor imaging” or DTI or “Diffusion-weighted imaging” or DWI* |
