# Supplementary material for: What are the neural correlates of meta-cognition and anosognosia in Alzheimer's disease? A systematic review
Source: Neurobiol Aging. 2020 Oct;94:250–64. doi: 10.1016/j.neurobiolaging.2020.06.011 (PMC7903321; doi:10.1016/j.neurobiolaging.2020.06.011)
Supplement: Supplementary Table 2 [file mmc2.docx]

| *Supplementary Table 2: Modified Newcastle-Ottawa quality assessment scale for included studies* | | | | | | | | | | | |
| --- | --- | --- | --- | --- | --- | --- | --- | --- | --- | --- | --- |
| Author (year) | Design | Selection | | | | | Comparability based on design and analysis | | Outcome | | Total score |
|  |  | Representative-ness of the sample | Sample size | Non-respondents | Ascertainment of exposure | |  |  | Assessment of the outcome | Statistical test |  |
| Amanzio et al., (2011) | Cross-sectional  Case-control | 🞣 |  |  | 🞣 | | 🞣 | | 🞣🞣 | 🞣 | 6  Satisfactory |
| Bertrand et al., (2018) | Cross-sectional  Case-control | 🞣 |  |  | 🞣 | | 🞣 | | 🞣🞣 | 🞣 | 6  Satisfactory |
| Berlingeri et al., (2015) | Cross-sectional  Case-control | 🞣 |  |  | 🞣 | | 🞣 | | 🞣🞣 | 🞣 | 6  Satisfactory |
| Consentino et al., (2015) | Cross-sectional  Case-control | 🞣 |  |  | 🞣 | | 🞣🞣 | | 🞣🞣 | 🞣 | 6  Satisfactory |
| De Castro et al., (2007) | Cross-sectional  correlational | 🞣 |  |  |  | | 🞣 | | 🞣🞣 |  | 4  Unsatisfactory |
| Derouesne et al., (1999) | Cross-sectional  Case-control | 🞣 |  |  | 🞣 | | 🞣 | | 🞣🞣 | 🞣 | 6  Satisfactory |
| Fujimoto et al., (2017) | Cross-sectional  correlational | 🞣 |  |  |  | | 🞣🞣 | | 🞣🞣 | 🞣 | 6  Satisfactory |
| Genon et al., (2014) | Cross-sectional  Correlational | 🞣 |  |  | 🞣 | | 🞣 | | 🞣🞣 | 🞣 | 6  Satisfactory |
| Author (year) | Design | Selection | | | | | Comparability based on design and analysis | | Outcome | | Total score |
|  |  | Representative of the sample | Sample size | Non-respondents | Ascertainment of exposure | |  |  | Assessment of the outcome | Statistical test |  |
| Genon et al., (2016) | Cross-sectional  correlational | 🞣 |  |  | 🞣 | |  | | 🞣🞣 | 🞣 | 5 Satisfactory |
| Guerrier et al., (2018) | Cross-sectional  correlational | 🞣 |  |  |  | | 🞣🞣 | | 🞣🞣 | 🞣 | 6 Satisfactory |
| Hanyu et al., (2008) | Cross-sectional  Case-control | 🞣 |  |  |  | |  | | 🞣🞣 | 🞣 | 4 Unsatisfactory |
| Harwood et al., (2005) | Cross-sectional  Correlational | 🞣 |  |  | 🞣 | | 🞣 | | 🞣🞣 | 🞣 | 6 Satisfactory |
| Hornberger et al., (2014) | Cross-sectional  correlational |  |  |  |  | |  | | 🞣🞣 |  | 2 Unsatisfactory |
| Jedidi et al., (2014) | Cross-sectional  Correlational | 🞣 |  |  |  | | 🞣🞣 | | 🞣🞣 | 🞣 | 6 Satisfactory |
| Massimo et al., (2013) | Cross-sectional  Correlational | 🞣 |  |  | 🞣 | | 🞣🞣 | | 🞣🞣 | 🞣 | 7  Good |
| Mimura & Yano (2006) | Cross-sectional  correlational | 🞣 |  |  | 🞣 | | 🞣 | | 🞣🞣 |  | 5 Satisfactory |
| Ott, Noto & Fogel (1996) | Cross-sectional  Correlational | 🞣 |  |  | 🞣 | | 🞣🞣 | | 🞣🞣 | 🞣 | 7  Good |
| Author (year) | Design | Selection | | | | | Comparability based on design and analysis | | Outcome | | Total score |
|  |  | Representative-ness of the sample | Sample size | Non-respondents | Ascertainment of exposure | |  |  | Assessment of the outcome | Statistical test |  |
| Perrotin et al., (2015) | Cross-sectional  Correlational | 🞣 |  |  | 🞣 | | 🞣🞣 | | 🞣🞣 | 🞣 | 7  Good |
| Philippi et al., (2017) | Cross-sectional  Case-control |  |  |  | 🞣 | |  | | 🞣🞣 |  | 3 Unsatisfactory |
| Rauchs et al., (2007) | Cross-sectional  correlational | 🞣 |  |  | 🞣 | |  | | 🞣🞣 | 🞣 | 5 Satisfactory |
| Reed, Jagust & Coulter (1993) | Cross-sectional  correlational | 🞣 |  |  | 🞣 | | 🞣 | | 🞣🞣 | 🞣 | 6 Satisfactory |
| Ruby et al., 2009 | Cross-sectional  correlational | 🞣 |  |  |  | |  | | 🞣🞣 | 🞣 | 4 Unsatisfactory |
| Salmon et al., 2006 | Cross-sectional  correlational | 🞣 | 🞣 |  |  | | 🞣 | | 🞣🞣 | 🞣 | 6 Satisfactory |
| Sedaghat et al., (2010) | Cross-sectional  Case-control | 🞣 |  |  | 🞣 | | 🞣 | | 🞣🞣 | 🞣 | 6 Satisfactory |
| Senturk et al., (2017) | Cross-sectional  Case-control | 🞣 |  |  | 🞣 | | 🞣 | | 🞣🞣 | 🞣 | 6  Satisfactory |
| Author (year) | Design | Selection | | | | | Comparability based on design and analysis | | Outcome | | Total score |
|  |  | Representative-ness of the sample | Sample size | Non-respondents | Ascertainment of exposure | |  |  | Assessment of the outcome | Statistical test |  |
| Shibata et al., (2008) | Cross-sectional  correlational | 🞣 |  |  |  | | 🞣 | | 🞣🞣 | 🞣 | 5  Satisfactory |
| Starkstein et al., (1995) | Cross-sectional  Case-control | 🞣 |  |  |  | | 🞣 | | 🞣🞣 | 🞣 | 5  Satisfactory |
| Sultzer et al., (2014) | Cross-sectional  Correlational | 🞣 |  |  | 🞣 | |  | | 🞣🞣 | 🞣 | 5  Satisfactory |
| Tagai et al., (2018) | Cross-sectional  Correlational and case-control | 🞣 |  |  |  | | 🞣🞣 | | 🞣🞣 | 🞣 | 6  Satisfactory |
| Tondelli et al., (2018) | Cross-sectional  correlational | 🞣 |  |  | 🞣 | | 🞣 | | 🞣🞣 | 🞣 | 6  Satisfactory |
| Vogel et al., (2005) | Cross-sectional  Correlational | 🞣 |  |  | 🞣 | | 🞣🞣 | | 🞣🞣 | 🞣 | 7  Good |
| Zamboni et al., (2013) | Cross-sectional  Correlational and case-control | 🞣 |  |  |  | | 🞣🞣 | | 🞣🞣 | 🞣 | 6  Satisfactory |
| 🞣 = 1 point  Points threshold: Very Good Studies: 9-10 points  Good Studies: 7-8 points | | | | | |  | | Satisfactory Studies: 5-6 points  Unsatisfactory Studies: 0 to 4 points | | | |
