# Supplementary material for: What are the neural correlates of meta-cognition and anosognosia in Alzheimer's disease? A systematic review
Source: Neurobiol Aging. 2020 Oct;94:250–64. doi: 10.1016/j.neurobiolaging.2020.06.011 (PMC7903321; doi:10.1016/j.neurobiolaging.2020.06.011)
Supplement: Supplementary Table 3 [file mmc3.docx]

| *Supplementary table 3: description of measurements and analysis used in structural imaging studies included in the systematic review* | | |  | |
| --- | --- | --- | --- | --- |
| Author (year) | Description of measurements | Type of memory | |  |
|  |  |  | |  |
|  | **Anosognosia** |  | |  |
| De Castro et al., (2007) | Self-consciousness questionnaire (SCQ) – **Patient/carer discrepancy score**:  14-item questionnaire self-appraisal questionnaire regarding insight that is verified by caregiver. The higher the score, the greater the self-consciousness (awareness)  Denial of illness scale (DIS) – **Clinician rated:**  Interview judged on 10 items. The higher the score on each item, the greater degree of anosognosia. | Anosognosia (no specific domain)  Anosognosia (no specific domain) | |  |
| Fujimoto et al., (2017) | Anosognosia Questionnaire adapted from Squire and Zouzounis – **Patient/carer discrepancy score**:  20-item questionnaire assessing awareness. The higher the discrepancy between the patient score and carer score, the higher the severity of anosognosia. | Anosognosia (no specific domain) | |  |
|  |  |  | |  |
| Guerrier et al., (2018) | Cognitive Difficulties Scale (CDS) – **Patient/carer discrepancy score:**  39-item questionnaire assessing concentration, memory and orientation completed by both patient with AD and caregivers. The higher the discrepancy between the patient score and carer score, the higher the severity of anosognosia. | Anosognosia (no specific domain) | |  |
| Hornberger et al., (2014) | Insight questionnaire – **Patient/carer discrepancy score**:  28-item insight questionnaire completed by patient and carer (answering regarding patient). The higher the discrepancy between the patient score and carer score, the higher the severity of anosognosia. | Anosognosia (no specific domain) | |  |
|  |  |  | |  |
| Ruby et al., 2009 | Judgment of personality - **Patient/carer discrepancy score**:  40 trait-adjectives based questions were used “are you sociable?”. These questions were framed to create 2 x 2 design. Patients were asked to take the first-person perspective and third person perspective of their self; whilst the carer reports on their perception of patient’s personality and what a third person may say. This created three different subtypes of anosognosia scores: self-judgement score, personality awareness score and third person perspective congruency score. Within each the higher the discrepancy between patients and carers answers, represented higher anosognosia scores. | Anosognosia (no specific domain) | |  |
| Senturk et al., (2017) | Anosognosia Questionnaire Dementia (AQ-D) – **Patient/carer discrepancy score**:  30 question assessing awareness. The higher the discrepancy between the patient score and carer score, the higher the severity of anosognosia.  Clinical Insight Rating Scale (CIRS) – **Clinician rated**:  5 questions regarding insight into their illness and day-to-day functional. Categorised into either “anosognosia” or “no anosognosia” | Anosognosia (no specific domain)  Anosognosia (no specific domain) | |  |
|  |  |  | |  |
|  | **Metacognition** |  | |  |
| Bertrand et al., (2018) | Episodic memory word task **Feeling of Knowing (FOK) Self-appraisal discrepancy scores**:  Feeling of knowing (FOK) task based on episodic memory retrieval task. The task requires participants with AD to remember twenty items across 4 trials of “pseudo trivia” (e.g. Cole Porter attended law school in Chicago. FOK is the discrepancy between predictive judgement and accurate retrieval scores formed the subject’s metacognition. The greater the discrepancy the lower the metacognitive ability. | Episodic Memory | |  |
| Consentino et al., (2015) | Episodic memory word task **Feeling of Knowing (FOK) Self-appraisal discrepancy scores**:  Feeling of knowing (FOK) task based on episodic memory retrieval task. The task requires participants with AD to remember twenty items across 4 trials of “pseudo trivia” (e.g. Cole Porter attended law school in Chicago. FOK is the discrepancy between predictive judgement and accurate retrieval scores formed the subject’s metacognition. The greater the discrepancy the lower the metacognitive ability. Metacognition reflected the ability to accurately judge when actual performance was high and where accuracy is low when predications are low. | Episodic Memory | |  |
| Genon et al., (2014) | Person descriptive adjectives - **Adapted Remember/Know Paradigm (Self-recognition task):**  The first stage of the procedure is the self-recognition task. 216 adjectives were used across nine runs. In each run during the encoding phase, one block of 15 words was allocated to self-relevance condition, and another block of words of 15 words allocate to other-relevance condition. This would be followed by a recognition phase using other random adjectives to see if participants can assign the correct adjectives to either self or other-condition. Whilst in a scanner, participants would push the button “yes” or “no” to recognising any words. The difference between correct and incorrect answers were monitored for self-accuracy and other-accuracy. Self-reference effect was the difference between “self-accuracy” and “other-accuracy”. SRE was predicted to be more impaired in participants with AD compared to healthy controls due to lower episodic memory performance.  Person descriptive adjectives **- Adapted Remember/Know Paradigm (Self-recollection task):**  The self-recollection task was performed 1-4 week(s) after the self-recognition task, in the participants home. The purpose of this task is to improve the accuracy of the R/K scoring from the first task and to provide qualitative/verbal reasoning for decision making by participant. Therefore, this task used a different set of 96 adjectives than the set used for the self-recognition task. The task adapted the Remember/know paradigm by training an experimenter to classify the participants subjective reports into the categories of R/K. If the participant provided any detail of relating the adjective to the encoding session 1-4 weeks previously then the experiment rated the answer as “remember”. If the participant reported they were familiar with the word but did not have any details relating to the encoding session, the experimenter rated the answer as “know”. | Episodic Memory  (Metacognition)  Episodic & Semantic Memory  (Metacognition) | |  |
| Genon et al., (2016) | Facial-name memory task **Feeling of Knowing (FOK)**  Feeling of knowing (FOK) task based on 85 full-face pictures of unfamous people (episodic memory) and 85 pictures of famous people (semantic memory). Participants were given a familiarisation phase to study the 85 unfamous faces before going into the encoding phase. The encoding phase associated full names with each image and participants were asked to immediately recall the name. After 10 minutes the participant moved to the prediction-recognition phase. During these phases the participants predicts how well they think they could correctly predict the full name, and then proceed to choose to select an answer from four options of the name associated with the picture. The discrepancy between predictive judgement and accurate retrieval scores formed the subject’s metacognition. The greater the discrepancy the lower the metacognitive ability. | Episodic & Semantic Memory | |  |
| Massimo et al., (2013) | Rey Complex Figure Immediate Free Recall – **Judgment of Learning (JOL):**  Self-discrepancy scores between actual performance and predictive performance on 1 task – Rey recall task. The ray recall task requires participants to look at a figure, perform an interference task for one minute, and then asked to draw figure from memory. The higher the discrepancy between the predictive score and actual score, the higher the severity of metamemory impairment | Visuospatial Working Memory | |  |
|  | **Both anosognosia and metacognition** |  | |  |
| Perrotin et al., (2015) | Cognitive Difficulties Scale (CDS) – **Patient/carer discrepancy score:**  39-item questionnaire assessing concentration, memory and orientation completed by both patient with AD and caregivers. The higher the discrepancy between the patient score and carer score, the higher the severity of anosognosia.  RL/RI-16 - **Judgement of Learning (JOLs):**  The Free and Cued Selective Reminding Test (RL/RI-16) is a 16-word verbal learning test of episodic memory. Minimal information surrounding procedure of RL/RI-16 was provided. Self-discrepancy scores between actual performance and predictive performance on 1 task (RL/RI-16). The higher the discrepancy between the predictive score and actual score, the higher the severity of metacognitive impairment. | Anosognosia (no specific domain)  Episodic Memory (Metacognition) | |  |
|  |  |  | |  |
| Philippi et al., (2017) | Word recognition memory test **- Remember/Know paradigm:**  Recognition memory test on 25 words that were provided during encoding. Then during recognition phase participants were asked to choose between the correct target word and a distractor. Each subject had to justify confidence in each answer: specifying if they “remembered”, “knew” or “guessed”. The greater the discrepancy between actual correct answers and judgements of whether answer is correct, the greater the severity of metacognitive impairment.  Study-specific Questionnaire – **Patient/carer discrepancy score:**  Clinician asked patient to answer five questions, which where then verified by carers. Score adjusted depending on carers report. The higher the score from the 5-likert based items, the more aware the patient was of their illness. | Anterograde memory  (Metacognition)  Anosognosia (no specific domain) | |  |
| Tondelli et al., (2018) | Anosognosia Questionnaire Dementia (AQ-D) – **Patient/carer discrepancy score**:  30 question assessing awareness. The higher the discrepancy between the patient score and carer score, the higher the severity of anosognosia.  Clinical Insight Rating Scale (CIRS) – **Clinician rated**:  5 questions regarding insight into their illness and day-to-day functional.  Self-appraisal discrepancies (SADS) – **Judgement of Learning (JOL)**:  Self-discrepancy scores between actual performance and predictive performance on 5 tasks – Babcock story recall, Rey auditory-verbal learning test, Rey complex figure task, Stroop test and frontal assessment battery. The higher the discrepancy between the predictive score and actual score, the higher the severity of metacognitive impairment. | Anosognosia (no specific domain)  Anosognosia (no specific domain)  Verbal Memory  Visuospatial Working Memory  Executive functions | |  |
