# Supplementary material for: What are the neural correlates of meta-cognition and anosognosia in Alzheimer's disease? A systematic review
Source: Neurobiol Aging. 2020 Oct;94:250–64. doi: 10.1016/j.neurobiolaging.2020.06.011 (PMC7903321; doi:10.1016/j.neurobiolaging.2020.06.011)
Supplement: Supplementary Table 4 [file mmc4.docx]

*Supplementary table 4: description of measurements and analysis used in functional imaging studies included in the systematic review*

| Author (year) | Description of measurements | Type of memory |  |
| --- | --- | --- | --- |
|  |  |  |  |
|  | **Anosognosia** |  |  |
| Amanzio et al., (2011) | Anosognosia Questionnaire Dementia (AQ-D) – **Patient/carer discrepancy score**:  30 question assessing awareness. The higher the discrepancy between the patient score and carer score, the higher the severity of anosognosia.  Clinical Insight Rating Scale (CIRS) – **Clinician rated**:  5 questions regarding insight into their illness and day-to-day functional.  Response Inhibition Task Assessment - **Go/No-go task**:  Participants viewed single uppercase letters in clear large font. Each stimulus appeared for 250ms with an interval of 1000ms to the next letter. The no-go stimulus was the letter X (frequency = 17%), meaning that the participant had to refrain from pushing the button after seeing the letter X. The participant was required to press the button for all other letters (go, frequency = 83%). The task was completed twice for familiarisation and then a third time during the MRI evaluation. | Anosognosia (no specific domain)  Anosognosia (no specific domain) | |
| De Castro et al., (2007) | Self-consciousness questionnaire (SCQ) – **Patient/carer discrepancy score:**  14-item questionnaire self-appraisal questionnaire regarding insight that is verified by caregiver. The higher the score, the greater the self-consciousness (awareness)  Denial of illness scale (DIS) – **Clinician rated:**  Interview judged on 10 items. The higher the score on each item, the greater degree of anosognosia. | Anosognosia (no specific domain)  Anosognosia (no specific domain) | |
| Derouesne et al., (1999) | Cognitive Difficulties Scale (CDS) – **Patient/carer discrepancy score:**  39-item questionnaire assessing concentration, memory and orientation completed by both patient with AD and caregivers. The higher the discrepancy between the patient score and carer score, the higher the severity of anosognosia.  Clinical interview – **Clinician rated:**  Investigator asks questions and categorises to “no anosognosia”, “mild anosognosia” and “severe anosognosia”.  Psycho-behavioural questionnaire (PBQ) – **Clinician rated:**  3 items relating to behavioural unawareness in every life from the 44-item PBQ questionnaire. | Anosognosia (no specific domain)  Anosognosia (no specific domain)  Anosognosia (no specific domain) | |
|  |  |  | |
| Guerrier et al., (2018) | Cognitive Difficulties Scale (CDS) – **Patient/carer discrepancy score:**  39-item questionnaire assessing concentration, memory and orientation completed by both patient with AD and caregivers. The higher the discrepancy between the patient score and carer score, the higher the severity of anosognosia. | Anosognosia (no specific domain) | |
| Hanyu et al., (2008) | Everyday Memory Checklist (EMC)- **Patient/carer discrepancy score:**  13-item questionnaire concerning areas of daily life completed by both patient with AD and caregivers. The higher the discrepancy between the patient score and carer score, the higher the severity of anosognosia. | Anosognosia (no specific domain) | |
| Harwood et al., (2005) | Neuro-behavioural rating (NRS) scale (insight item score) – **Clinician rated:**  NRS item 12 (insight question) is answered by patient. The patient’s self-assessment is them compared to objective cognitive impairment and interviews with the caregiver. Clinician judgement – scored from 0 “insight preserved” to 6 “unaware of deficits” | Anosognosia (no specific domain) | |
| Jedidi et al., (2014) | Judgment of personality - **Patient/carer discrepancy score:**  40 personality trait adjectives. i.e. “currently, am I aggressive?” The higher the discrepancy between the patient score and carer score of the patient, the higher the severity of anosognosia. | Anosognosia (no specific domain) | |
|  |  |  | |
| Ott, Noto & Fogel (1996) | Clinical Insight Rating Scale – **Clinician rated**:  5 questions regarding insight into their illness and day-to-day functional (Instrumental Activities of Daily Living). | Anosognosia (no specific domain) | |
|  |  |  | |
|  |  |  | |
|  |  |  | |
| Reed, Jagust & Coulter (1993) | Anosognosia rating scale – **Clinician rated:**  Clinician interviews participant and provides overall impression by placing participant into one of four categories: “full awareness”, “shallow awareness”, “no awareness” or “denies impairment”. | Anosognosia (no specific domain) | |
| Ruby et al., 2009 | Judgment of personality - **Patient/carer discrepancy score**:  40 trait-adjectives based questions were used “are you sociable?”. These questions were framed to create 2 x 2 design. Patients were asked to take the first-person perspective and third person perspective of their self; whilst the carer reports on their perception of patient’s personality and what a third person may say. This created three different subtypes of anosognosia scores: self-judgement score, personality awareness score and third person perspective congruency score. Within each the higher the discrepancy between patients and carers answers, represented higher anosognosia scores. | Anosognosia (no specific domain) | |
| Salmon et al., 2006 | Self-made anosognosia questionnaire - **Patient/carer discrepancy score**:  questionnaire to assess 13 cognitive domains regarding memory and awareness. The higher the discrepancy between the patient score and carer score, the higher the severity of anosognosia. | Anosognosia (no specific domain) | |
| Sedaghat et al., (2010) | Clinical interview – **Clinician rated:**  Clinician interviews patient and carer. Placed participants into one of two groups: “Anosognosia” or “no anosognosia” | Anosognosia (no specific domain) | |
| Shibata et al., (2008) | Anosognosia Questionnaire adapted from Squire and Zouzounis – **Patient/carer discrepancy score**:  20-item questionnaire assessing awareness. The higher the discrepancy between the patient score and carer score, the higher the severity of anosognosia. | Anosognosia (no specific domain) | |
| Starkstein et al., (1995) | Anosognosia Questionnaire Dementia – **Patient/carer discrepancy score**:  30 question assessing awareness. The higher the discrepancy between the patient score and carer score, the higher the severity of anosognosia. | Anosognosia (no specific domain) | |
| Sultzer et al., (2014) | Neuro-behavioural rating (NRS) scale (insight item score) – **Clinician rated:**  NRS item 12 (insight question) is answered by patient. The patient’s self-assessment is them compared to objective cognitive impairment and interviews with the caregiver. Clinician judgement | Anosognosia (no specific domain) | |
| Tagai et al., (2018) | Anosognosia Questionnaire Dementia – **Patient/carer discrepancy score**:  30 question assessing awareness. The higher the discrepancy between the patient score and carer score, the higher the severity of anosognosia. | Anosognosia (no specific domain) | |
| Vogel et al., (2005) | Anosognosia rating scale – **Clinician rated:**  Clinician interviews participant and provides overall impression by placing participant into one of four categories: “full awareness”, “shallow awareness”, “no awareness” or “denies impairment”.  Memory questionnaire – **Patient/carer discrepancy score**:  20-item questionnaire on memory abilities. The higher the discrepancy between the patient score and carer score, the higher the severity of anosognosia. | Anosognosia (no specific domain) | |
| Zamboni et al., (2013) | Anosognosia Questionnaire Dementia – **Patient/carer discrepancy score**:  30 question assessing awareness. The higher the discrepancy between the patient score and carer score, the higher the severity of anosognosia.  Anderson Trait List Activation - **Self and other Paradigm** (**Patient/carer discrepancy score)**:  discrepancy scores between patient and carer scores on questions based on Anderson traits list (36 adjectives-based questions. Each adjective presented in two conditions: “self” and “other” – which related to their relative. Whilst in the scan, participants provided “yes” or “no” answers the question. E.g. “are you forgetful?” and compared to carers who completed paper form of questionnaire. The higher the discrepancy score the higher the severity of anosognosia. | Anosognosia (no specific domain) | |
|  |  |  | |
|  | **Metacognition** |  | |
| Genon et al., (2014) | Person descriptive adjectives - **Adapted Remember/Know Paradigm (Self-recognition task):**  The first stage of the procedure is the self-recognition task. 216 adjectives were used across nine runs. In each run during the encoding phase, one block of 15 words was allocated to self-relevance condition, and another block of words of 15 words allocate to other-relevance condition. This would be followed by a recognition phase using other random adjectives to see if participants can assign the correct adjectives to either self or other-condition. Whilst in a scanner, participants would push the button “yes” or “no” to recognising any words. The difference between correct and incorrect answers were monitored for self-accuracy and other-accuracy. Self-reference effect was the difference between “self-accuracy” and “other-accuracy”. SRE was predicted to be more impaired in participants with AD compared to healthy controls due to lower episodic memory performance.  Person descriptive adjectives **- Adapted Remember/Know Paradigm (Self-recollection task):**  The self-recollection task was performed 1-4 week(s) after the self-recognition task, in the participants home. The purpose of this task is to improve the accuracy of the R/K scoring from the first task and to provide qualitative/verbal reasoning for decision making by participant. Therefore, this task used a different set of 96 adjectives than the set used for the self-recognition task. The task adapted the Remember/know paradigm by training an experimenter to classify the participants subjective reports into the categories of R/K. If the participant provided any detail of relating the adjective to the encoding session 1-4 weeks previously then the experiment rated the answer as “remember”. If the participant reported they were familiar with the word but did not have any details relating to the encoding session, the experimenter rated the answer as “know”. | Episodic Memory  (Metacognition)  Episodic & Semantic Memory  (Metacognition) | |
| Mimura & Yano (2006) | Auditory Verbal Learning Test (AVLT) - **Judgment of Learning (JOLs)**:  AVLT uses a 15-item list of words for participants to study and remember (3-4 syllables). Then the recollection task incorporated provide the first list and a distractor list. Two sessions of AVLT were completed. Judgement of Learning was accumulated: (1) Post-diction 1 after completing the 1^st^ session (2) provided actual performance score for session 1 (3) Prediction for the 2^nd^ session (3) (4) post-diction for the second session (5) actual performance score for session 2. Self-discrepancy scores between actual performance and predictive performance on one task – The higher the discrepancy between the predictive score and actual score, the higher the severity of metacognitive impairment. | Episodic Memory  (Metacognition) | |
| Rauchs et al., (2007) | Episodic memory word task **- Remember/ Know paradigm**:  Assessed episodic memory using Grober and Buscke’s procedure. Encoding phase for participants was to remember 15 words in 5 successive trails. The design is to limit semantic memory impairment. After learning, the episodic memory task was 4-6 hours later. The 15 words are presented in series of three (5 rounds). The participant will point out and read aloud each item in response to the examiner naming the category for each round. For example, category might be “vegetable” and the 3 items are “carrot”, “peas” and “sweetcorn”. Once all series of three cards are recalled correctly, go onto the next series. This is the encoding phase. The recollection phase comes after a night’s sleep (12-hour delay) and with the introduction of 15 semantic and 15 neutral distractors. Yes/no recognition task using a remember/know paradigm is implemented. “Remember” (R) is retrieval accompanied by the recollection of specific experiences present at encoding, or “Know” (K) is retrieval achieved on feeling of familiarity. Impairment to episodic memory is typically associated with more correct K responses and less R responses. | Episodic Memory  (Metacognition) | |
|  | **Both anosognosia and metacognition** |  | |
|  |  |  | |
| Berlingeri et al., (2015) | Anosognosia Questionnaire Dementia (AQ-D) - **Patient/carer discrepancy score**:  30-item questionnaire assessing awareness. The higher the discrepancy between the patient score and carer score, the higher the severity of anosognosia.  Episodic word recognition task - **Judgment of Learning (JOLs)**:  Forced choice word recognition task using 30 three-syllable words that were paired with a distractor word. There was a study phase and recollection phase. Judgment of learning scores were accumulated over 4 phases (1) prediction phase – before the task. (2) comparison phase – after completing task, assess whether their level was lower, equal or higher than they had predicated (3) Post-diction phase – specific quantitative estimate of how many answers they got correct. (4) follow up – after 30 minutes, presented again with prediction table. The higher the discrepancy between the predictive score and actual score, the higher the severity of metacognitive impairment.  Semantic Verbal Fluency Test - **Judgment of Learning (JOLs)**:  Recall as many names of fruit as possible in 1 minute. Judgment of learning scores were accumulated over 4 phases (1) prediction phase – before the task. (2) comparison phase – after completing task, assess whether their level was lower, equal or higher than they had predicated (3) Post-diction phase – specific quantitative estimate of how many answers they got correct. (4) follow up – after 30 minutes, presented again with prediction table. The higher the discrepancy between the predictive score and actual score, the higher the severity of metacognitive impairment. | Anosognosia (no specific domain)  Episodic memory  (Metacognition)  Semantic Memory  (Metacognition) | |
|  |  |  | |
| Perrotin et al., (2015) | Cognitive Difficulties Scale (CDS) – **Patient/carer discrepancy score:**  39-item questionnaire assessing concentration, memory and orientation completed by both patient with AD and caregivers. The higher the discrepancy between the patient score and carer score, the higher the severity of anosognosia.  RL/RI-16 - **Judgement of Learning (JOLs):**  The Free and Cued Selective Reminding Test (RL/RI-16) is a 16-word verbal learning test of episodic memory. Minimal information surrounding procedure of RL/RI-16 was provided. Self-discrepancy scores between actual performance and predictive performance on 1 task (RL/RI-16). The higher the discrepancy between the predictive score and actual score, the higher the severity of metacognitive impairment. | Anosognosia (no specific domain)  Episodic Memory (Metacognition) | |
| Philippi et al., (2017) | Word recognition memory test **- Remember/Know paradigm:**  Recognition memory test on 25 words that were provided during encoding. Then during recognition phase participants were asked to choose between the correct target word and a distractor. Each subject had to justify confidence in each answer: specifying if they “remembered”, “knew” or “guessed”. The greater the discrepancy between actual correct answers and judgements of whether answer is correct, the greater the severity of metacognitive impairment.  Study-specific Questionnaire – **Patient/carer discrepancy score**:  Clinician asked patient to answer five questions, which were then verified by carers. Score adjusted depending on carers report. The higher the score from the 5-likert based items, the more aware the patient was of their illness. | Anterograde memory  (Metacognition)  Anosognosia (no specific domain) | |
|  |  |  | |
